# Validation of Different Nutritional Assessment Tools in Predicting Prognosis of Patients with Soft Tissue Spindle-Cell Sarcomas

**DOI:** 10.3390/nu10060765

**Published:** 2018-06-13

**Authors:** Hiromi Sasaki, Satoshi Nagano, Setsuro Komiya, Noboru Taniguchi, Takao Setoguchi

**Affiliations:** 1 Department of Orthopaedic Surgery, Graduate School of Medical and Dental Sciences, Kagoshima University, Kagoshima 890-8520, Japan; piro422@m2.kufm.kagoshima-u.ac.jp (H.S.); naga@m2.kufm.kagoshima-u.ac.jp (S.N.); skomiya@m3.kufm.kagoshima-u.ac.jp (S.K.); tanigu@m2.kufm.kagoshima-u.ac.jp (N.T.); 2 Department of Medical Joint Materials, Graduate School of Medical and Dental Sciences, Kagoshima University, Kagoshima 890-8520, Japan

**Keywords:** soft tissue sarcomas (STS), Glasgow prognostic score (GPS), Geriatric Nutritional Risk Index (GNRI), neutrophil–lymphocyte ratio (NLR), platelet–lymphocyte ratio (PLR), controlling nutritional (CONUT) score, prognosis

## Abstract

Predicting outcomes in patients with soft tissue sarcoma (STS) is challenging. To improve these predictions, we retrospectively analyzed common nutritional assessment systems, including Glasgow prognostic score (GPS), Geriatric Nutritional Risk Index (GNRI), neutrophil–lymphocyte ratio (NLR), platelet–lymphocyte ratio (PLR), and controlling nutritional (CONUT) score against outcomes in 103 patients with STS, of whom 15 (14.6%) died within 1 year of diagnosis. GPS, GNRI, NLR, PLR, and CONUT scores significantly differed between patients who died within one year and patients who lived longer. Binomial logistic regression analysis showed that male sex, older age at diagnosis, higher GPS, higher stage, and unresectable STS were risk factors for death within a year of diagnosis. Overall survival was evaluated by Cox proportional hazards models, which correlated higher NLR, higher PLR, larger maximum diameter of tumor, higher stage, and unresectable STS with poor prognosis. We next examined prognostic factors in the 93 patients with resectable STS, and found male sex, higher GPS, and higher stage were correlated with poor prognosis in these patients. Our findings suggest that GPS, NLR, and PLR are simple predictors of outcome in patients with STS. Nutritional therapies might improve their GPS and prognosis.

## 1. Introduction

Soft tissue sarcomas (STS) are a heterogeneous group of tumors composed of more than 50 histological subtypes, that affect almost every site in the body and retain the full range of malignant behavior [1]. In Japan, the most common histologic categories are undifferentiated pleomorphic sarcoma (UPS), well differentiated liposarcoma (WDLS), myxoid/round cell liposarcoma (MRLS), leiomyosarcoma (LMS), myxofibrosarcoma (MFS), and synovial sarcoma [2]. Their most common site among Japanese patients is the lower extremities in patients with UPS, WDLS, MRLS, LMS, MFS, and synovial sarcoma [2]. Older age, male sex, deep tumor location, and onset at the trunk or neck are associated with significantly poorer prognosis [2]. Their treatment should be tailored by the patient’s age, site of onset, clinicopathologic subtype, staging, and comorbidity [3]. Predicting precise outcomes and chances of cure in patients with STS is difficult.

High rates of malnutrition (40–80%) have been reported in cancer patients [4]. Malnutrition is a main cause of poor prognosis [5]. Many nutritional examination tools including weight loss, body mass index, blood chemical analysis, and body composition are used to predict cancer prognoses, leading to inconsistent results [6,7,8,9]. The Geriatric Nutritional Risk Index (GNRI) is generated to examine risk of malnutrition-related complications in elderly patients [10,11], and is reportedly a significant predictor of prognosis in many diseases including cancer [12,13,14,15,16,17,18,19]. The Glasgow prognostic score (GPS) was developed to help predict cancer outcomes [20]. The neutrophil–lymphocyte ratio (NLR) and platelet–lymphocyte ratio (PLR) can predict prognosis of several cancer types [21,22,23,24,25,26]. The controlling nutritional score (CONUT) is a predictor of heart failure and cancer and is based on two biochemical parameters (serum albumin and cholesterol level), and one immune parameter (total lymphocyte count) to examine nutritional status [27,28,29,30,31].

A consideration with these nutritional assessment tools is that they are inadequate when used alone, whereas several tools have been combined in some studies for more sensitive and specific assessments of nutritional status [32,33]. To improve prediction of prognosis in patients with STS, we evaluated their correlations with one-year and overall survival in a group of patients with STS.

## 2. Subjects and Methods

### 2.1. Patients’ Data

We retrospectively reviewed records of 103 patients who were treated for STS (spindle cell sarcoma) at the Department of Orthopedic Surgery, Kagoshima University, from January 2007 to December 2014. Patients’ clinical characteristics were collected from medical records, including sex, age, height, weight, routine pre-operative blood test, histological types, tumor size, location, stage, treatment, surgical margins, and survival times. Patients for whom some of these data were missing were excluded from the study. Nutritional assessments were calculated from patients’ clinical data.

### 2.2. Geriatric Nutritional Risk Index

GNRI was calculated from serum albumin and body weight using the following formula: GNRI = [1.489 × albumin (g/L)] + [41.7 × (body weight/ideal body weight)]. Body weight or ideal body weight were set to 1 when the patient’s body weight exceeded the ideal body weight [10]. The ideal body weight was defined as a body mass index of 22 [13,34].

### 2.3. Glasgow Prognostic Score

GPS was derived by allocating one point each for elevated C-reactive protein (CRP) (>10 mg/L) and hypoalbuminemia (<3.5 mg/L), so that patients with both, one, or none of these conditions would have scores of 2, 1, or 0, respectively [20].

### 2.4. Neutrophil–Lymphocyte Ratio

NLR is calculated from neutrophil and lymphocyte counts, as previously reported [21].

### 2.5. Platelet–Lymphocyte Ratio

PLR is calculated from lymphocyte and platelet counts, as previously reported [24].

### 2.6. Controlling Nutritional Score

CONUT score is calculated from serum albumin concentration, lymphocyte count, and total cholesterol concentration, as previously reported [31].

### 2.7. Statistical Analysis

Patients were divided into those who died within 1 year of their diagnoses (shorter-lived group), and those who lived more than 1 year after diagnosis (longer-lived group). Difference of variables between the longer- and shorter-lived groups were examined by Student’s *t* test, Mann–Whitney U test, Fisher’s exact test, and Cochran–Armitage test. Multivariable stepwise binomial logistic regression analysis and the Cox proportional hazards model were used to evaluate relationships between prognosis and variables. Correlation coefficients were analyzed by Spearman’s rank correlation coefficient. When correlation coefficients between variables were >0.6, only the variable with the highest correlation with survival time was selected. Because of the relatively small number of patients and the large number of variables, we applied a stepwise variable selection method to identify significant factors, as previously described [35]. *p* < 0.05 was considered significant. Analysis was performed using add-in software, BellCurve for Excel (Social Survey Research Information Co., Ltd., Tokyo, Japan).

### 2.8. Ethics Approval and Consent to Participate

The study protocol was approved by the institutional review board of Kagoshima University and was in accordance with the 1964 Helsinki declaration and its later amendments or comparable ethical standards. The patients were informed that their medical data would be submitted for publication and gave their consent to do so.

## 3. Results

**Figure 1:**
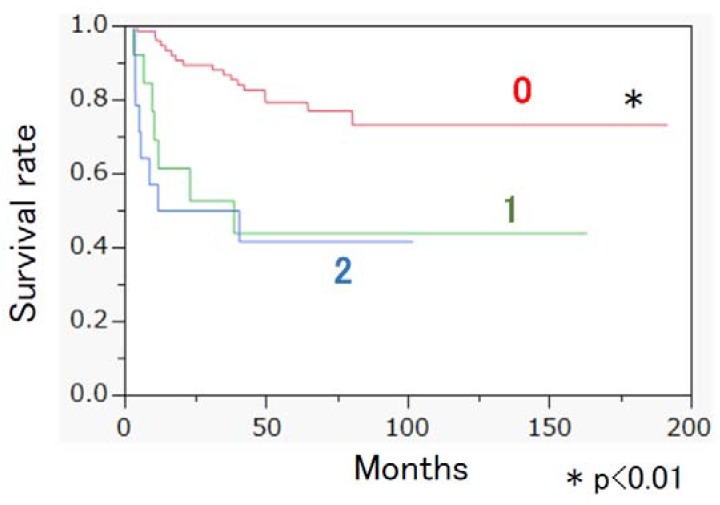
Kaplan–Meier survival analysis of each Glasgow prognostic score (GPS) group; Kaplan–Meier survival analysis showed that the GPS 0 group showed significantly longer median survival time than did the GPS 1 and GPS 2 groups.

The clinical and demographic characteristics of the 103 patients with STS are shown in Table 1. Histologic categories, UICC TNM staging, type of treatment, and surgical margins are shown in the supplemental data. The rate of death within one year following their STS diagnoses was 14.6% (15/103). Age at diagnosis, GPS, GNRI, NLR, PLR, and CONUT score, maximum tumor diameter, stage, and proportion of resectable STS differed significantly between the longer- and shorter-lived groups (Table 2). Binomial logistic regression analysis showed that male sex, older age at diagnosis, higher GPS, higher stage, and unresectable STS were risk factors for the shorter-lived groups (Table 3). Kaplan–Meier survival analysis showed that the GPS 0 group showed significantly longer median survival time than did the GPS 1 and GPS 2 groups (Figure 1). Risk factors for overall survival were examined by the Cox proportional hazards model. Higher NLR, higher PLR, larger maximum diameter of tumor, higher stage, and unresectable STS were correlated with poor prognosis (Table 4). We next examined prognostic factors in the patients with resectable STS (Table 5). The Cox proportional hazards model showed that male sex, higher GPS, and higher stage were correlated with poor prognosis in patients with resectable STS (Table 6).

## 4. Discussion

Patient-related factors, such as weight loss, low nutritional status, systemic inflammation, and decreased immunity can affect prognosis of patients with malignant tumor [36,37]. As Forrest et al. developed GPS as a prognostic score based on the combination of an inflammation marker (CRP) and a nutritional marker (hypoalbuminemia) [20,38], GPS is considered to reflect nutritional status [39,40]. GPS is derived by allocating one point each for elevated CRP (>10 mg/L) and hypoalbuminemia (<3.5 mg/L), so that patients with both, one, or none of these conditions would have scores of 2, 1, or 0, respectively [20]. However, for the modified GPS (mGPS), patients with hypoalbuminemia were assigned a score of 0 in the absence of an elevated C-reactive protein [41]. Because serum albumin is a commonly used marker for diagnosing malnutrition, we used GPS rather than mGPS [39,42] in this study. Although NLR and PLR are primarily considered to be indicators of inflammation, they are also regarded as nutritional indicators because total lymphocyte counts are included in nutritional screening tools [43,44] and malnutrition and immune suppression are closely associated with one another [45]. Nutritional and inflammation status are difficult to separate because malnutrition and inflammation coexist as part of a two-way causal malnutrition-inflammation cycle, in which malnutrition increases risk and severity of inflammation, and inflammation impairs nutritional status by decreasing food intake and impairing micronutrient absorption [46,47,48]. We examined whether nutritional assessment tools, including GPS, GNRI, NLR, PLR, and CONUT scores, were associated with prognosis in patients with STS, and found that GPS, GNRI, NLR, PLR, and CONUT scores differed significantly between longer- and shorter-lived patients. These findings suggest that these groups’ nutritional status is significantly different. It is the chicken or the egg dilemma, with significantly different nutritional status and STS resulting in death within a year of diagnosis. Although this question is not clarified, these different variables are prognostic factors of death within 1 year of diagnosis.

Logistic regression analysis also associated higher GPS with the shorter-lived group. GPS is reportedly correlated with prognosis in various types of malignancy, independent of age, stage, or performance status [36,49,50,51,52,53,54]. Nakamura et al. also reported that the high-sensitivity modified Glasgow prognostic score (Hs-mGPS), which uses 3 mg/L (rather than 10 mg/L) as the CRP cut-off value, can help predict survival of patients with STS [55]. Our logistic regression analysis showed that the combination of sex, age, GPS, stage, and resectable vs unresectable gave the highest coefficient of determination (*R*^2^ = 0.640). These findings indicate that combining GPS with other variables can improve the accuracy of prognosis prediction for clinicians who treat STS. In addition, as GPS is calculated by the combination of serum CRP and albumin, improvement of albumin by nutritional support might improve prognosis of patients with STS. Our Cox proportional hazards model correlated higher NLR and higher PLR with poorer overall survival in patients with STS. NLR and PLR are reported to be prognostic factors in patients with STS [56,57,58,59]. Lymphocytes can affect tumor growth and metastasis via endogenous anti-cancer immune activity [60], whereas neutrophils promote progression of cancer through the production of cytokines [61] and immune suppression [62]. Platelets promote tumor growth and metastasis [63]. High pre-operative PLR was associated with poorer prognosis in STS [59]. Our findings suggest that NLR and PLR, and the combination of maximum tumor diameter, stage, and resectability improve prognosis prediction in patients with STS.

Prognostic markers are useful in selecting patients who could benefit from chemotherapy and radiation following resection. In addition, treatment of these prognostic factors might improve patient adherence to adjuvant therapy. In this regard, we evaluated factors associated with prognosis in STS patients who underwent resections. Our Cox proportional hazards model correlated higher GPS with poor prognosis in patients with resectable STS. These findings suggest that adjuvant therapy should be considered for high-risk patients following resections.

All cancer patients should be screened for malnutrition, and substrate and energy requirements should be met by step-wise nutritional interventions, from counseling to parenteral nutrition [64]. More than 70 nutritional assessment tools have been reported in different populations [65]. Although nutritional screening is recommended, no fully sensitive and specific nutritional assessment tool has been established [66]. Our findings showed that GPS, NLR, and PLR are prognostic nutritional markers in STS patients. A strength of our paper is the use of multiple nutritional assessment tools to evaluate prognosis of STS patients. Nutritional support should be considered for these high-risk patients. Therapies for cancer-associated malnutrition include nutritional counselling, oral nutritional supplements, artificial nutrition, physical therapy, and drug therapy [64].

Our study has several limitations. First, this study was a single-center cohort study, so selection bias may have occurred. A multicenter study should be performed to check these findings. Second, we tested relatively few patients and variables; a larger study with a bigger cohort is required to accurately assess risk factors. We did not examine outpatient nutritional intake; patients’ dietary habits should be examined more comprehensively. Patients also had a broad range of histological diagnoses.

## 5. Conclusions

Nutritional evaluation tools, including GPS, NLR, and PLR, are clinically convenient predictors of outcomes in patients with STS, and can help predict prognoses and improve management of Japanese patients with STS. Our findings also show that male sex, older age at diagnosis, higher GPS, higher stage, and unresectability are risk factors for death within one year. Complementary nutritional therapies might improve the GPS and prognosis of high-risk patients with STS.

## Figures and Tables

**Table 1:** Demographic data of soft tissue sarcoma (STS) patients.

Variables	
Proportion of female	48.5% (50/103)
Diagnosis age	64 (52–73)
WBC (/µL)	6060 (4855–7530)
Plate (×10^4^/µL)	24.5 (20.1–29.4)
T-cholesterol (mg/dL)	191.5 ± 39.1
GPS	0.0 (0.0–1.0)
GNRI	102.7 (95.3–107.5)
NLR	2.3 (1.6–3.3)
PLR	15.0 (12.3–19.2)
CONUT score	1.0 (0.0–2.0)
Maximum diameter of tumor	70.0 (48.5–100.0)
Proportion of trunk onset	20.4% (21/103)
Stage (cases)	2 (32); 3 (60); 4 (11)
Proportion of resectable STS	90.3% (93/103)
Survival time (months)	60.6 ± 39.6
Survival rate at one year	85.4% (88/103)

Abbreviations: WBC: white blood cell; GNRI: geriatric nutritional risk index; GPS: Glasgow prognostic score; NLR: neutrophil–lymphocyte ratio; PLR: platelet–lymphocyte ratio; CONUT score: controlling nutritional status score; STS: soft tissue sarcoma.

**Table 2:** Difference of variables between patients who died within one year and patients who lived longer.

Factor	Death within 1 Year	1 Year Survival	*p* Value
Number	15	88	
Proportion of female	40.0% (6/15)	50.0% (44/88)	0.581
Diagnosis age	72 (64.5–81.5)	64 (51.0–70.5)	0.009 **
WBC (/µL)	6220 (5570–9005)	5950 (4783–7393)	0.217
Plate (×10^4^/µL)	26.3 (21.9–30.9)	24.2 (19.8–28.6)	0.231
T- cholesterol (mg/dl)	204.1 ± 35.7	189.3 ± 39.4	0.178
GPS	1.0 (1.0–2.0)	0.0 (0.0–0.0)	<0.001 **
GNRI	89.3 (86.0–95.3)	104.2 (98.2–108.7)	<0.001 **
NLR	4.0 (2.6–5.8)	2.2 (1.6–3.0)	0.003 **
PLR	19.6 (15.5–26.7)	14.6 (11.7–17.7)	0.003 **
CONUT score	3.0 (2.0–4.5)	1.0 (0.0–2.0)	<0.001 **
Maximum diameter of tumor	112.0 (94.0–150.0)	65.5 (40.8–94.3)	<0.001 **
Proportion of trunk onset	26.7% (4/15)	19.3% (17/88)	0.50
Stage (cases)	1(0)/2(1)/3(9)/4(5)	1(0)/2(31)/3(51)/4(6)	0.002 **
Proportion of resectable STS	53.3% (8/15)	96.6% (85/88)	<0.001 **

Abbreviations: WBC: white blood cell; GNRI: geriatric nutritional risk index; GPS: Glasgow prognostic score; NLR: neutrophil–lymphocyte ratio; PLR: platelet–lymphocyte ratio; CONUT score: controlling nutritional status score; STS: soft tissue sarcoma; ** *p* < 0.01

**Table 3:** Binomial logistic regression analysis for the risk factor of death within one year.

Coefficient of Determination *R*^2^:0.640		
Variables	HR (95% CI)	*p* Value
Female	0.074 (0.006–0.974)	0.048 *
Diagnosis age	1.090 (1.009–1.177)	0.030 *
GPS	8.660 (1.986–37.245)	0.004 **
NLR	1.368 (0.842–2.221)	0.206
Stage	27.512 (1.974–383.486)	0.014 *
Resectable STS	0.010 (0.001–0.175)	0.002 **

Abbreviations: HR-hazard ratio; GPS: Glasgow prognostic score; NLR: neutrophil–lymphocyte ratio; STS: soft tissue sarcoma; * *p* < 0.05; ** *p* < 0.01

**Table 4:** Risk factors for poor prognosis of patients with STS.

	Cox Proportional Hazards Model	
	HR (95% CI)	*p* Value
NLR	1.229 (1.032–1.462)	0.020 *
PLR	1.016 (1.002–1.031)	0.028 *
Maximum diameter of Tumor	1.004 (1.001–1.007)	0.006 **
Stage	2.779 (1.424–5.422)	0.003 **
Resectable STS	0.131 (0.051–0.338)	<0.001 **

Abbreviations: NLR: neutrophil–lymphocyte ratio; PLR: platelet–lymphocyte ratio; STS: soft tissue sarcoma; * *p* < 0.05; ** *p* < 0.01

**Table 5:** Demographic data of STS patients with resection surgery.

Variables	
Proportion of female	47.3% (44/93)
Diagnosis age	64 (51–73)
WBC (/µL)	6110 (4900–7600)
Plate (×10^4^/µL)	24.9 (20.2–29.5)
GPS	0.0 (0.0–0.0)
GNRI	104.2 (96.8–108.5)
NLR	2.3 (1.6–3.2)
PLR	15.0 (11.8–19.0)
CONUT score	1.0 (0.0–2.0)
Maximum diameter of tumor	69.0 (42.0–100.0)
Proportion of trunk onset	18.3% (17/93)
Proportion of deep onset	47.3% (44/93)
Stage (cases)	2 (31); 3 (55); 4 (7)
Survival time (months)	65.4 ± 38.2

Abbreviations: WBC: white blood cell; GNRI: geriatric nutritional risk index; GPS: Glasgow prognostic score; NLR: neutrophil–lymphocyte ratio; PLR: platelet–lymphocyte ratio; CONUT score: controlling nutritional status score.

**Table 6:** Risk factors for poor prognosis of STS patients with resection surgery.

	Cox Proportional Hazards Model	
	HR (95% CI)	*p* Value
Female	0.313 (0.128–0.767)	0.011 *
Age	1.024 (0.993–1.055)	0.126
GPS	2.098 (1.299–3.388)	0.002 **
Trunk onset	0.316 (0.073–1.375)	0.125
Stage	3.336 (1.405–7.924)	0.006 **

* *p* < 0.05; ** *p* < 0.01; Abbreviations: GPS: Glasgow prognostic score.
